# Risk of Peripheral Artery Disease and Venous Thromboembolism in Patients with Neuromyelitis Optic Spectrum Disorder: A Nationwide Population-Based Cohort Study

**DOI:** 10.7150/ijms.130836

**Published:** 2026-07-22

**Authors:** Xin-An Chen, Chun-Nan Wu, Tung-Han Tsai, Chien-Yu Lin, Hsuan Chang, Shuo-Yan Gau, Kuang-Hua Huang, Chien-Ying Lee

**Affiliations:** 1 School of Medicine, Chung Shan Medical University, Taichung 40201, Taiwan.; 2 Department of Pharmacology, Chung Shan Medical University, Taichung 40201, Taiwan.; 3 Department of Pharmacy, Chung Shan Medical University Hospital, Taichung 40201, Taiwan.; 4 Department of Health Services Administration, China Medical University, Taichung 406040, Taiwan.; 5 Department of Medical Education, Ditmanson Medical Foundation Chia-Yi Christian Hospital, Chiayi City 600566, Taiwan.

**Keywords:** neuromyelitis optic spectrum disorder, peripheral artery disease, venous thromboembolism

## Abstract

**Background:**

The relationship between Neuromyelitis optica spectrum disorder (NMOSD) and specific cardiovascular outcomes, particularly macrovascular events such as peripheral artery disease (PAD) and venous thromboembolism (VTE), has not been examined in a population-based study. The current study investigated the association between NMOSD and the risks of PAD and VTE.

**Materials and Methods:**

This retrospective cohort study used data from the Taiwan National Health Insurance Research Database. Patients with new-onset NMOSD between 2003 and 2020. Patients with previous PAD or VTE were excluded. Each patient was matched to five general patients for comparison using propensity score matching. In total, the study included 2,027 patients with NMOSD and 10,135 matched general population controls. A Cox proportional hazards model was used to investigate PAD and VTE risk, with relevant variables controlled for. NMOSD was stratified by severity to further verify the association between disease severity and macrovascular event risk. The control variables considered in this study were sex, age, insured salary, urbanization, Charlson comorbidity index (CCI), and related comorbidities.

**Results:**

The average follow-up of all participants was 7.01 person-years. After adjustments for relevant variables, patients with NMOSD had significantly higher risks of PAD (adjusted hazard ratio [aHR] = 1.40; 95% confidence interval [CI]: 1.02-1.92) and VTE (aHR = 4.81; 95% CI: 3.08-7.52). The risk of vascular events was strongly associated with disease severity. Severe NMOSD was associated with markedly increased risks of PAD (aHR = 2.26; 95% CI: 1.41-3.61) and VTE (aHR = 11.69; 95% CI: 6.86-19.90), whereas mild and moderate disease did not significantly increase PAD risk.

**Conclusion:**

NMOSD is a significant risk factor for PAD and VTE. These findings highlight the importance of proactive vascular risk assessment and preventive management in patients with NMOSD.

## Introduction

Neuromyelitis optica spectrum disorder (NMOSD), previously known as Devic's disease, is a rare autoimmune astrocytopathy of the central nervous system, and its prevalence is 0.5-4.4 per 100,000 individuals 1, 2. The disorder is characterized by recurrent inflammatory attacks mediated by pathogenic autoantibodies that primarily target aquaporin-4 (AQP4) water channels on astrocytic foot processes. This immune response leads to complement-dependent astrocytic injury, blood-brain barrier disruption, and subsequent demyelination and axonal loss 3-5. Emerging evidence further indicates that AQP4-IgG-mediated complement activation 6 not only damages astrocytes but also amplifies perivascular inflammation and recruits granulocytes, thereby extending injury beyond the central nervous system microenvironment 7. NMOSD most frequently affects the optic nerves and spinal cord, presenting as recurrent optic neuritis and longitudinally extensive transverse myelitis. Additional core features include area postrema syndrome and involvement of the brainstem, diencephalon, and cerebrum 1, 2, 4.

In addition to its neurological manifestations, NMOSD may increase the risk of thromboembolic events through autoimmune and immobility-related pathways. Systemic autoimmune activity and chronic inflammation can trigger endothelial activation and prothrombotic changes, including increased tissue factor expression, elevated plasminogen activator inhibitor-1 levels, impaired protein C anticoagulant activity, and complement-mediated endothelial injury, all of which promote hypercoagulability 8-12. Recent studies also suggest that inflammatory cytokine cascades and neutrophil extracellular trap (NET) formation may further potentiate coagulation activation and endothelial dysfunction in NMOSD, providing a more integrated immunothrombotic framework 7, 13. Moreover, neurological disability and recurrent relapses often lead to hospitalization and reduced mobility, creating venous stasis and further increasing the risk of VTE in patients with NMOSD 8, 9.

Autoimmune diseases such as systemic lupus erythematosus (SLE), inflammatory bowel disease (IBD), and Behçet's syndrome are strongly associated with VTE, which underscores the broader vascular effects of immune-mediated inflammation 10-12. Persistent inflammation and complement activation may also contribute to arterial injury and atherosclerotic progression, suggesting a mechanistic link between NMOSD and peripheral arterial disease (PAD) 14. In particular, chronic immune-mediated endothelial injury, oxidative stress, and dysregulated vascular repair mechanisms have been implicated in accelerating atherosclerosis in autoimmune conditions, which may extend to NMOSD despite limited direct evidence 13. Although studies have reported that coexisting autoimmune diseases are frequently observed in patients with NMOSD 15-17, direct epidemiological evidence quantifying thromboembolic risk in NMOSD remains limited. In particular, the association between NMOSD and PAD has not been systematically evaluated. Furthermore, current literature has primarily focused on venous thromboembolism, while the potential contribution of NMOSD-related immune dysregulation to arterial pathology remains underexplored, highlighting a critical knowledge gap 7, 13. Therefore, this cohort study used the Taiwan National Health Insurance Research Database (NHIRD) to examine the risks of VTE and PAD in patients with NMOSD and determine whether NMOSD independently contributes to macrovascular complications beyond conventional risk factors.

## Materials and Methods

### Data Source

This study conducted a secondary analysis by using data from the NHIRD for the period between 2002 and 2022. The NHIRD, managed by Taiwan's Health and Welfare Data Science Center (HWDC), is a comprehensive population-based database that contains outpatient and inpatient medical records for beneficiaries of the National Health Insurance (NHI) program. To ensure confidentiality, the HWDC replaces all personal identifiers with encrypted, randomly generated codes. The NHI was launched in 1995, and it currently provides health coverage to nearly 99% of Taiwan's residents. The NHIRD is therefore widely used as a real-world data source for clinical research and health policy studies 18, 19. In the NHIRD, diseases were coded in accordance with the *International Classification of Diseases, Ninth Revision, Clinical Modification* (*ICD-9-CM*) system before 2016, and subsequently, they were coded using the *International Classification of Diseases, Tenth Revision, Clinical Modification* (*ICD-10-CM*) system.

### Ethics Statements

For this study, all data were fully anonymized to protect the privacy of beneficiaries, and informed consent was not required because the dataset contained only deidentified information. The research was conducted in compliance with the principles of the Declaration of Helsinki and followed the STROCSS reporting guidelines. Ethical approval for the study protocol was obtained from the Institutional Review Board of Chung Shan Medical University Hospital, Taiwan (CSMUH CS1-24227).

### Study Participants

**Figure 1:**
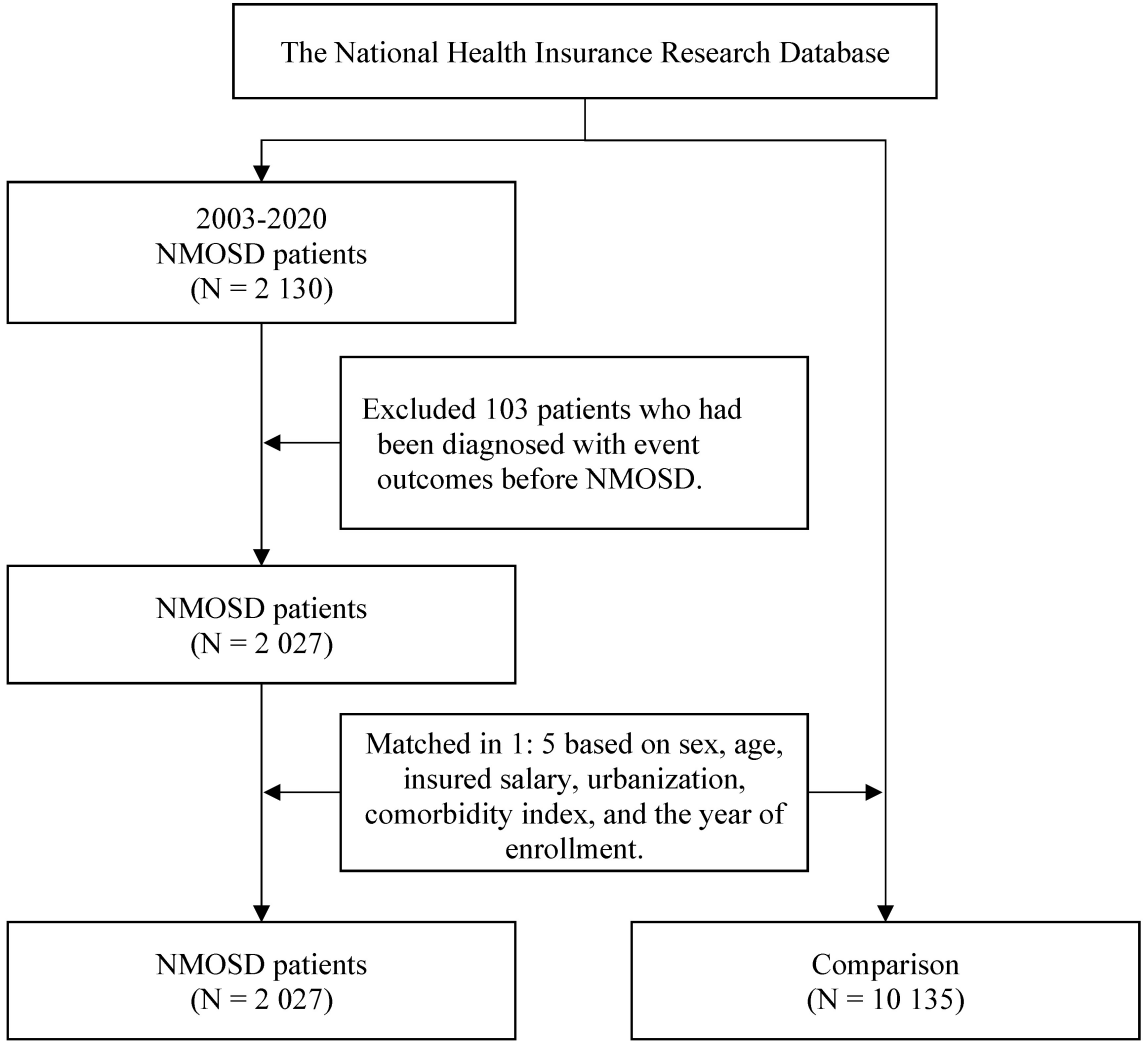
The flowchart of participant selection. Abbreviation: NMOSD, neuromyelitis optica spectrum disorder.

This study identified patients with a new diagnosis of NMOSD between 2003 and 2020. The case definition for NMOSD (*ICD-9-CM* 341.0; *ICD-10-CM* G36.0) required at least three outpatient visits with an NMOSD diagnosis within 1 year. Patients with a previous diagnosis of PAD or VTE recorded before the diagnosis of NMOSD were excluded. To reduce selection bias arising from imbalanced covariates in observational studies, we conducted propensity score matching at a 1:5 ratio. Propensity scores were estimated using logistic regression, and a probability value was generated for each patient with NMOSD on the basis of their individual characteristics. This approach enabled balanced comparisons and reduced confounding. The matched variables included sex, age, insured salary, urbanization level, Charlson comorbidity index (CCI), and year of cohort entry. After matching, the study included 2,027 patients with NMOSD and 10,135 matched general population controls. The patient selection process is presented in **Figure 1**.

### Study Design

In this retrospective cohort study, we defined the incidence of PAD (*ICD-9-CM 443.9; ICD-10-CM I73.9*) and VTE based on at least one inpatient diagnosis or three or more outpatient diagnosis. VTE was identified through codes for deep venous thrombosis (DVT; *ICD-9-CM 453.4; ICD-10-CM I82.4*) and pulmonary embolism (PE; *ICD-9-CM 415.1; ICD-10-CM I26*). For the NMOSD group, follow-up began on the date of diagnosis. Matched controls were assigned the same index date as their corresponding NMOSD cases. Each participant was followed from the index date until death, a new diagnosis of PAD or VTE, or the study endpoint in 2022, whichever occurred first. The analysis also accounted for comorbidities, including hypertension (*ICD-9-CM* 401-405; *ICD-10-CM* I10-I13 and I15), diabetes mellitus (*ICD-9-CM* 250; *ICD-10-CM* E08-E13), hyperlipidemia (I*CD-9-CM* 272; *ICD-10-CM* E78), anxiety (*ICD-9-CM* 300.0; *ICD-10-CM* F40-F41), and sleep disturbance (*ICD-9-CM* 780; *ICD-10-CM* G47.9). These comorbidities were identified via the outpatient and inpatient diagnoses of each participant in the year before the index date.

### Statistical Analysis

All statistical analyses were conducted using SAS software, version 9.4 (SAS Institute, Cary, NC, USA). A p value of <0.05 was considered significant. The standardized mean differences were used to compare the distribution differences of baseline characteristics between the NMOSD and control groups. A standardized mean difference of <0.1 was regarded as negligible. A Cox proportional hazards model was used to investigate the risks of PAD and VTE in patients with NMOSD after adjustment for all relevant variables. The results are presented as hazard ratios (HRs) with 95% confidence intervals (CIs). To ensure the validity of the Cox proportional-hazards model, we tested the proportional hazards assumption using graphical assessment (log-log plots) and found no significant violations. Furthermore, to account for the potential confounding effect of death during the long-term follow-up, competing risk analyses were conducted to estimate the hazard ratios for PAD and VTE events. A subgroup analysis was performed to examine the association between NMOSD severity and the risks of PAD and VTE. NMOSD severity was measured as the proportion of total hospital stay attributable to NMOSD during the follow-up period divided by the complete follow-up duration. NMOSD severity was categorized as mild (first tertile), moderate (second tertile) and severe (third tertile). Furthermore, stratified analyses by sex and age were performed to evaluate the consistency of the association between NMOSD and the risks of PAD and VTE across different clinical subgroups.

## Results

A total of 12,162 individuals were enrolled in the study, including 2,027 patients with NMOSD and 10,135 matched controls. **Table 1** summarizes their baseline characteristics. The NMOSD cohort had a higher proportion of female (73.5%; n = 1,490). The mean ages of the patients in the NMOSD and control cohorts were 43.46 ± 16.42 and 45.74 ± 20.23 years, respectively. The patient with NMOSD and matched controls were well balanced in terms of sex, age, insured salary, urbanization level, and CCI score, as evidenced by standardized mean differences all being less than 0.1. However, the patients with NMOSD had higher prevalences of hyperlipidemia (8.44% vs. 5.18%), anxiety (9.47% vs. 4.28%), and sleep disturbance (14.16% vs. 7.58%) than did the matched controls.

**Table 2** presents the incidence of PAD in the patients with NMOSD and matched controls. During follow-up, the cumulative incidence of PAD in the NMOSD group was 2.52%, which was higher than the rate of 1.77% observed in the controls. The incidence rate of PAD was 3.77 per 1,000 person-years in the NMOSD group and 2.50 per 1,000 person-years in the control group. In the multivariable Cox regression analysis, NMOSD was independently associated with an increased risk of PAD (adjusted HR [aHR] = 1.40; 95% CI = 1.02-1.92). The competing risk analysis also revealed that the NMOSD group had a higher risk of incident PAD (aHR = 1.27; 95% CI = 1.01-1.79). The sex-specific incidence rates of PAD were 2.87 and 2.22 per 1,000 person-years in female and male, respectively. Sex was not a significant predictor of PAD (male vs. female, aHR = 0.84; 95% CI = 0.61-1.15). Age was a significant predictor of PAD; a higher risk of PAD was observed among individuals aged 35-44 years (aHR = 2.81; 95% CI = 1.25-6.28) and those aged ≥45 years (aHR = 5.61; 95% CI = 2.59-12.15).

**Figure 2:**
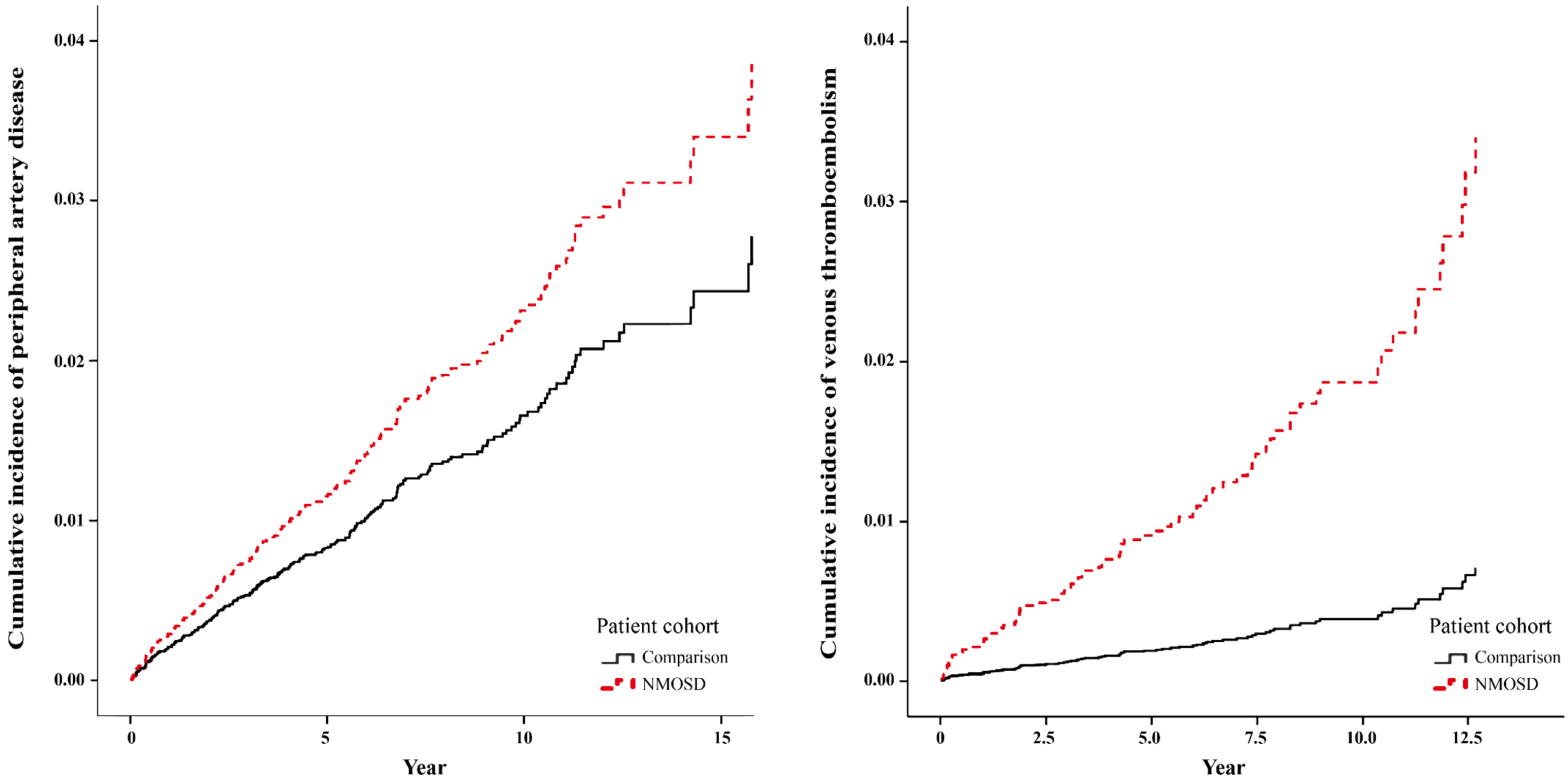
Cumulative incidence curve of macrovascular events in patients with NMOSD (log-rank test, p <0.001). Abbreviation: NMOSD, neuromyelitis optica spectrum disorder.

**Table 3** presents the incidence of VTE in patients with NMOSD and matched controls. During follow-up, the cumulative incidence of VTE in the NMOSD group was 1.87%, which was higher than the rate of 0.41% observed in the controls. The incidence rate of VTE was 0.28 per 1,000 person-years in the NMOSD group and 0.06 per 1,000 person-years in the control group. The results of the multivariable analysis revealed that NMOSD was strongly associated with a higher risk of VTE (aHR = 4.81; 95% CI = 3.08-7.52). The competing risk analysis also revealed that the NMOSD group had a higher risk of incident VTE (aHR = 4.49; 95% CI = 2.90-6.97). The sex-specific incidence rates were 0.11 and 0.05 per 1,000 person-years for female and male, respectively. In sex-adjusted analysis, male had a lower risk of VTE than did female (aHR = 0.49; 95% CI = 0.26-0.90). The risk of VTE also increased with age, particularly among individuals aged ≥45 years (aHR = 6.17; 95% CI = 1.46-25.98). **Figure 2** presents the cumulative incidence curves of PAD and VTE, which reveal a significantly higher incidence in patients with NMOSD than in matched controls (log-rank test, p <0.001).

**Table 4** presents the findings of subgroup analysis stratified by NMOSD severity. The risk of PAD was significantly higher in the severe NMOSD subgroup (aHR = 2.26; 95% CI = 1.41-3.61), whereas the mild (aHR = 1.50; 95% CI = 0.99-2.27) and moderate subgroups (aHR = 0.49; 95% CI = 0.20-1.19) did not demonstrate a significant increase in the risk of PAD. For VTE, including deep venous thrombosis (DVT) and pulmonary embolism (PE), both the moderate and severe NMOSD subgroups exhibited substantially elevated risks compared with controls, indicating that increasing disease severity is strongly associated with higher VTE incidence. The results of the stratified analysis are presented in **Table 5**. In terms of PAD, the increased risk associated with NMOSD was particularly prominent and statistically significant in male patients (aHR = 1.97; 95% CI = 1.04-3.74). Although higher aHRs were observed in younger age groups (e.g., <45 years), these did not reach statistical significance. For VTE, NMOSD was associated with a significantly higher risk across most subgroups. Notably, female with NMOSD exhibited a more than 5-fold increase in VTE risk compared to the comparison group (aHR = 5.21; 95% CI = 3.21-8.46). When stratified by age, the risk of VTE was substantially elevated in all groups aged 25 and older, with the highest magnitude observed in the 35-44 years age group (aHR = 9.01; 95% CI = 3.01-26.97).

## Discussion

In this population-based cohort study, patients with NMOSD had higher risks of both PAD and VTE, including DVT and PE. The risk of DVT was particularly high in these patients. Older age further increased the risks of PAD and VTE. In addition, disease severity affected outcomes: the severe NMOSD subgroup had the highest risk of PAD, whereas the moderate and severe NMOSD subgroups had a high risk of VTE. Furthermore, male had a significantly lower risk of VTE than did female.

The increased thromboembolic risk in NMOSD may be related to the combined effects of systemic autoimmune activity and neurological disability. Specifically, it has been proposed that inflammatory responses in NMOSD can disrupt vascular integrity and alter coagulation pathways, creating a prothrombotic environment 8, 9. From a systemic immunopathological perspective, NMOSD is characterized by AQP4-IgG-mediated inflammatory cascades accompanied by peripheral immune dysregulation, including cytokine release and immune cell alterations, which play a central role in disease progression 7, 20. Rather than being confined to the central nervous system, these immune processes may also contribute to endothelial dysfunction and coagulation imbalance.

In particular, proinflammatory cytokines and activated innate immune cells have been implicated as key mediators of disease activity 20, and may simultaneously promote a prothrombotic milieu. Circulating inflammatory markers such as neutrophil-to-lymphocyte ratio (NLR) and platelet-to-lymphocyte ratio (PLR), which are associated with disease activity and relapse in NMOSD, may further reflect this sustained systemic inflammatory state 7. Moreover, recurrent relapses often necessitate hospitalization, and the resulting periods of reduced mobility promote venous stasis, thereby increasing the risk of VTE 8, 9.

Autoimmune disorders are well-established risk factors for VTE. Conditions such as SLE, IBD, and Behçet's syndrome are associated with an increased risk of VTE 10. Several studies have expanded on this, demonstrating that a wide range of autoimmune and immune-mediated diseases are associated with increased risks of both PE and VTE 9-12. Notably, sex-related differences have also been observed among autoimmune conditions. Unlike immune-mediated conditions such as coeliac disease and Hashimoto's thyroiditis, in which male have a disproportionately higher risk of VTE, as indicated by our findings, male patients with NMOSD have a significantly lower risk of VTE than female patients with NMOSD do 21. Several mechanisms have been proposed to explain this association, including chronic systemic inflammation, increased tissue factor expression, elevated plasminogen activator inhibitor-1 levels, and impaired protein C anticoagulant activity; all of these contribute to a prothrombotic state 12. These processes may be further potentiated by persistent endothelial dysfunction, which has been increasingly recognized as a central mediator linking inflammation and thrombosis in autoimmune diseases. Additionally, emerging evidence suggests that systemic inflammatory markers, such as elevated neutrophil-to-lymphocyte ratio and increased B-cell activity, are associated with disease activity and may indirectly reflect a prothrombotic state in NMOSD 13. Furthermore, hospitalization is a risk factor because individuals admitted for autoimmune or immune-mediated disorders are more likely to develop subsequent VTE 12, 21.

With respect to PAD, our results suggest a possible association between NMOSD and an increased risk of PAD. Rather than a direct disease-specific effect, this association may reflect cumulative vascular injury driven by chronic inflammation, endothelial dysfunction, and impaired vascular repair, which are known contributors to atherosclerotic progression in systemic autoimmune conditions 13. In addition, our analysis identified hypertension and depression as significant comorbid factors associated with PAD risk in NMOSD. This finding may be explained by the persistent systemic inflammatory milieu characteristic of NMOSD, as evidenced by elevated peripheral inflammatory markers such as C-reactive protein and neutrophil-to-lymphocyte ratio, which have also been implicated in atherosclerotic progression 7, 22, 23. Furthermore, inflammatory activity reflected by markers such as NLR and B-cell activation has been shown to correlate with disease severity and relapse in NMOSD, suggesting a sustained inflammatory burden that may potentiate vascular injury 13, 23. Within this context, the coexistence of hypertension and depression may further amplify inflammation-mediated endothelial dysfunction, thereby contributing to PAD development. This observation is partially consistent with previous findings indicating that cardiovascular conditions in MS, NMOSD, and transverse myelitis are as common as they are in the general population, indicating a need for more detailed characterization of the vascular risks in these diseases 14. Taken together, these findings suggest a potential mechanistic link between chronic inflammation and vascular injury in NMOSD; however, direct mechanistic evidence in NMOSD remains limited, and further studies are warranted to elucidate the underlying pathophysiological mechanisms.

Our findings reveal a significantly higher incidence of VTE in the patients with NMOSD than in the control population. This finding is consistent with that of a previous study indicating that patients NMOSD may have an increased risk of VTE 24. From a mechanistic perspective, in addition to neurologic disability leading to venous stasis, autoimmune and inflammatory mechanisms may contribute to hypercoagulability and elevated VTE risk in NMOSD 24. Importantly, these mechanisms are likely synergistic, with systemic inflammation enhancing coagulation activation while endothelial injury reduces antithrombotic capacity, thereby lowering the threshold for thrombosis 7, 13. Such interactions may reflect an integrated immunothrombotic process rather than isolated pathological pathways 25. In this study, analysis revealed a positive correlation between VTE incidence and increasing age, suggesting that both NMOSD-related factors and age-associated conditions contribute to the increased VTE risk.

We observed that the risks of both DVT and PE—together constituting VTE 10—were significantly higher in the patients with NMOSD than in the general population, with DVT contributing most prominently to this increase. Consistent with our findings, previous studies have reported that VTE is a common complication in NMOSD, especially among individuals with TM 25. In addition, VTE occurred in 12.0% of patients with NMOSD during acute attacks in our cohort, which closely aligns with a previously reported rate of 10.9% 24. This temporal association with acute disease activity further supports the role of inflammation-driven thrombogenicity. These results highlight the increased susceptibility of patients with NMOSD to severe thrombotic events.

On the other hand, we determined that patients with NMOSD had an increased risk of PAD, with this particularly true for those aged ≥45 years. However, the current analysis did not demonstrate a significant association between female sex and a higher PAD risk. Given these inconsistencies, further investigation is warranted to clarify the nature and strength of this association.

We observed that patients with NMOSD had an increased risk of VTE, which is consistent with previous evidence indicating that cardiovascular and treatment-related factors contribute to thrombotic complications in this population 24. The risk of VTE was also significantly higher among individuals aged ≥45 years; this finding is consistent with that of a previous study indicating that advanced age and prolonged hospitalization are associated with an increased risk of VTE in NMOSD 8. These results indicate that VTE risk in NMOSD is multifactorial and affected by comorbidities, age, sex, and factors related to treatment or immobilization 24.

We noted that the risk of PAD was associated with NMOSD severity, with the highest risk observed in individuals with severe NMOSD. One possible interpretation is that greater disease severity reflects a higher cumulative inflammatory burden, which may accelerate vascular remodeling and atherosclerotic processes over time 13. Given that cardiovascular comorbidities and related risk factors are common and associated with poor outcomes in demyelinating disorders such as myelin oligodendrocyte glycoprotein antibody-associated disease and double seronegative NMOSD 26, our findings suggest that patients with NMOSD have a different risk profile outside the inpatient setting. Moreover, although hospitalized patients with NMOSD generally have prolonged lengths of stay compared with nonhospitalized patients 6, PAD risk did not appear to be disproportionately elevated in this subgroup.

Existing research suggests that NLR and PLR are significantly correlated with NMOSD severity and pathophysiology, reflecting systemic immune-inflammatory states with high clinical utility 22, 27, 28. In this study, NMOSD severity was operationalized using hospitalization duration and categorized into tertiles as a pragmatic proxy, given the absence of standardized clinical measures—such as the Expanded Disability Status Scale (EDSS), relapse frequency, and MRI findings—in the NHIRD. Although hospitalization duration has been used in administrative database research as an indirect indicator of disease burden, it does not fully capture the multidimensional nature of NMOSD severity, including neurological disability progression, subclinical disease activity, and relapse severity. Consequently, misclassification may arise, particularly if hospitalization duration is influenced by non-disease-related factors such as healthcare access, physician practice patterns, or comorbidities. This non-differential misclassification may attenuate true associations and bias subgroup estimates toward the null; therefore, findings from severity-stratified analyses should be interpreted with caution.

We determined that the risk of VTE increased with NMOSD severity, with the highest risk observed in patients with moderate to severe disease. This pattern is consistent with previous evidence indicating that hospitalization for immune-mediated diseases increases the risk of VTE 21. This gradient of risk supports a model in which thrombotic complications are driven not only by immobilization but also by the intensity of systemic inflammation and its downstream vascular effects. Such findings reinforce the hypothesis that inflammatory burden plays a central role in thrombotic susceptibility in NMOSD 7. These findings highlight the importance of early identification and proactive management of VTE risk in this vulnerable population.

Our study has several notable strengths. Few studies have examined the relationship between NMOSD and PAD and VTE using HRs. We extend this work by identifying the associated risks, whereas previous studies have primarily focused on their prevalence. We used data from nationwide population-based cohorts in Taiwan, ensuring that our large sample is highly representative and provides strong statistical precision. The NHIRD includes data for nearly the entire Taiwanese population, providing a solid foundation for population-based research. Its comprehensive coverage also helps mitigate selection bias, which is a common drawback in observational studies. Although prospective population-based cohort studies are often preferred for identifying risk factors, our retrospective cohort study, using detailed insurance data available in the NHIRD, can be considered a dependable and effective alternative design.

This study has several limitations that should be acknowledged. First, the dataset did not contain key information on established thromboembolic risk factors in NMOSD, including smoking status, alcohol consumption, body mass index, physical activity, personal medical history, disease activity, and disease duration. Second, in this study, NMOSD severity was proxied by the proportion of total hospital stay, representing the cumulative clinical burden. However, we acknowledge several limitations: disease-specific characteristics such as AQP4 antibody status, precise relapse frequency, and the specific use of immunosuppressive or biologic therapies could not be directly captured. These factors may exert complex effects on systemic inflammation and vascular function. While our severity definition (based on LOS tertiles) serves as a functional surrogate in a nationwide database, the lack of information on treatment exposure and temporal changes in disease activity may introduce residual confounding. Future research with integrated clinical registries is warranted to refine these severity assessments. Furthermore, all diagnoses were identified using *ICD-9-CM* and *ICD-10-CM* codes. Although Taiwan's Bureau of National Health Insurance performs random chart audits to maintain coding accuracy, the presence of unmeasured confounders cannot be fully excluded.

## Conclusion

NMOSD is associated with a significantly increased risk of both PAD and VTE, with the risk of VTE being particularly elevated. Older age and greater disease severity further contribute to these risks. Notably, in this study, the risk of VTE was significantly higher in hospitalized patients, whereas the risk of PAD was higher among nonhospitalized individuals. Additionally, male patients had a lower risk of VTE than their female counterparts did. These findings underscore the need for timely vascular risk assessment and proactive management in patients with NMOSD.

## Figures and Tables

**Table 1:** Distribution of baseline characteristics after matching.

Variables	Total		Comparison		NMOSD ^b^		SMD ^b^
	N	%	N	%	N	%	
Total	12,162	100.00	10,135	100.00	2,027	100.00	
Sex ^a^							
Female	8,942	73.52	7,452	73.53	1,490	73.51	0.000
Male	3,220	26.48	2,683	26.47	537	26.49	0.000
Age (year) ^a^							
<25	1,742	14.32	1,460	14.41	282	13.91	-0.014
25-34	2,083	17.13	1,736	17.13	347	17.12	0.000
35-44	2,944	24.21	2,454	24.21	490	24.17	-0.001
≥45	5,393	44.34	4,485	44.25	908	44.80	0.011
Mean ± SD	45.36 ± 19.66		45.74 ± 20.23		43.46 ± 16.42		
Insured salary (NTD) ^a^							
≤21,000	3,266	26.85	2,722	26.86	544	26.84	0.000
21,001-24,000	3,254	26.76	2,712	26.76	542	26.74	0.000
24,001-40,100	2,752	22.63	2,295	22.64	457	22.55	-0.002
≥40,001	2,890	23.76	2,406	23.74	484	23.88	0.003
Urbanization ^a^							
High	7,987	65.67	6,645	65.56	1,342	66.21	0.014
Medium	3,404	27.99	2,845	28.07	559	27.58	-0.011
Low	771	6.34	645	6.36	126	6.22	-0.006
CCI score ^a, b^							
0	5,084	41.80	4,230	41.74	854	42.13	0.008
1	2,771	22.78	2,315	22.84	456	22.50	-0.008
≥2	4,307	35.41	3,590	35.42	717	35.37	-0.001
Year of inclusion							
2003	186	1.53	155	1.53	31	1.53	0.000
2004	126	1.04	105	1.04	21	1.04	0.000
2005	174	1.43	145	1.43	29	1.43	0.000
2006	102	0.84	85	0.84	17	0.84	0.000
2007	174	1.43	145	1.43	29	1.43	0.000
2008	216	1.78	180	1.78	36	1.78	0.000
2009	402	3.31	335	3.31	67	3.31	0.000
2010	444	3.65	370	3.65	74	3.65	0.000
2011	462	3.80	385	3.80	77	3.80	0.000
2012	738	6.07	615	6.07	123	6.07	0.000
2013	840	6.91	700	6.91	140	6.91	0.000
2014	972	7.99	810	7.99	162	7.99	0.000
2015	1,008	8.29	840	8.29	168	8.29	0.000
2016	1,182	9.72	985	9.72	197	9.72	0.000
2017	1,236	10.16	1,030	10.16	206	10.16	0.000
2018	1,212	9.97	1,010	9.97	202	9.97	0.000
2019	1,374	11.30	1,145	11.30	229	11.30	0.000
2020	1,314	10.80	1,095	10.80	219	10.80	0.000
Comorbidities							
Hypertension							
No	10,355	85.14	8,640	85.25	1,715	84.61	-0.018
Yes	1,807	14.86	1,495	14.75	312	15.39	0.018
Diabetes mellitus							
No	10,634	87.44	8,790	86.73	1,844	90.97	0.135
Yes	1,528	12.56	1,345	13.27	183	9.03	-0.135
Hyperlipidemia							
No	11,466	94.28	9,610	94.82	1,856	91.56	-0.130
Yes	696	5.72	525	5.18	171	8.44	0.130
Anxiety							
No	11,536	94.85	9,701	95.72	1,835	90.53	-0.206
Yes	626	5.15	434	4.28	192	9.47	0.206
Sleep disturbance							
No	11,107	91.33	9,367	92.42	1,740	85.84	-0.213
Yes	1,055	8.67	768	7.58	287	14.16	0.213

^a^ Matching variable. ^b^ Abbreviation: NMOSD, neuromyelitis optica spectrum disorder; SMD, standardized mean difference; CCI, Charlson comorbidity index.

**Table 2:** The incidence rate and risk of incident peripheral artery disease.

Variables	Peripheral artery disease					
	Events N	%	Follow-up year	IR ^a^	Adjusted HR (95% CI)	p-value
Total	230	1.89	85,213	2.70		
Patient cohort						
Comparison	179	1.77	71,671	2.50	Reference	
NMOSD ^a^	51	2.52	13,542	3.77	1.40 (1.02-1.92)	0.039
Sex						
Female	181	2.02	63,159	2.87	Reference	
Male	49	1.52	22,054	2.22	0.84 (0.61-1.15)	0.273
Age (year)						
<25	7	0.40	12,856	0.54	Reference	
25-34	17	0.82	16,030	1.06	1.73 (0.72-4.19)	0.223
35-44	40	1.36	22,387	1.79	2.81 (1.25-6.28)	0.012
≥45	166	3.08	33,940	4.89	5.61 (2.59-12.15)	<0.001
Insured salary (NTD)						
≤21,000	86	2.63	27,815	3.09	Reference	
21,001-24,000	56	1.72	19,472	2.88	0.82 (0.59-1.16)	0.271
24,001-40,100	42	1.53	18,882	2.22	0.82 (0.56-1.19)	0.298
≥40,001	46	1.59	19,044	2.42	0.77 (0.53-1.11)	0.158
Urbanization						
High	142	1.78	55,578	2.55	Reference	
Medium	66	1.94	23,884	2.76	1.15 (0.76-1.72)	0.511
Low	22	2.85	5,751	3.83	1.08 (0.66-1.76)	0.760
CCI score ^a^						
0	45	0.89	37,225	1.21	Reference	
1	64	2.31	20,506	3.12	1.81 (1.23-2.67)	0.003
≥2	121	2.81	27,482	4.40	2.00 (1.37-2.91)	<0.001
Comorbidities						
Hypertension	74	4.10	11,045	6.70	1.56 (1.16-2.10)	0.004
Diabetes mellitus	53	3.47	9,213	5.75	1.21 (0.87-1.69)	0.252
Hyperlipidemia	24	3.45	4,525	5.30	1.07 (0.70-1.65)	0.750
Anxiety	33	5.27	4,490	7.35	1.92 (1.31-2.81)	<0.001
Sleep disturbance	59	5.59	9,482	6.22	1.98 (1.46-2.70)	<0.001

^a^ Abbreviation: IR, incident rate per 1,000 person-year; NMOSD, neuromyelitis optica spectrum disorder; CCI, Charlson comorbidity index.

**Table 3:** The incidence rate and risk of incident venous thromboembolism.

Variables	Venous thromboembolism					
	Events N	%	Follow-up year	IR ^a^	adjusted HR (95% CI)	p-value
Total	80	0.66	86,129	0.09		
Patient cohort						
Comparison	42	0.41	72,399	0.06	Reference	
NMOSD ^a^	38	1.87	13,730	0.28	4.81 (3.08-7.52)	<0.001
Sex						
Female	68	0.76	63,889	0.11	Reference	
Male	12	0.37	22,241	0.05	0.49 (0.26-0.90)	0.023
Age (year)						
<25	3	0.06	12,886	0.16	Reference	
25-34	9	0.28	16,098	0.56	2.95 (0.63-13.74)	0.169
35-44	15	0.55	22,577	0.66	3.48 (0.79-15.30)	0.099
≥45	53	1.00	34,568	0.16	6.17 (1.46-25.98)	0.013
Insured salary (NTD)						
≤21,000	24	0.73	28,260	0.09	Reference	
21,001-24,000	23	0.71	19,603	0.12	1.42 (0.79-2.55)	0.244
24,001-40,100	18	0.65	19,037	0.10	1.19 (0.64-2.23)	0.578
≥40,001	15	0.52	19,229	0.08	0.89 (0.46-1.71)	0.724
Urbanization						
High	52	0.65	56,165	0.93	Reference	
Medium	23	0.68	24,120	0.95	1.22 (0.60-2.55)	0.208
Low	5	0.65	5,844	0.86	1.02 (0.38-2.79)	0.964
CCI score ^a^						
0	18	0.35	37,388	0.05	Reference	
1	18	0.65	20,757	0.09	1.46 (0.75-2.85)	0.261
≥2	44	1.02	27,985	0.16	2.27 (1.25-4.13)	0.007
Comorbidities						
Hypertension	25	1.38	11,382	0.22	1.70 (1.01-2.84)	0.045
Diabetes mellitus	16	1.05	9,384	0.17	1.14 (0.63-2.07)	0.657
Hyperlipidemia	10	1.44	4,602	0.22	1.32 (0.67-2.59)	0.430
Anxiety	8	1.28	4,664	0.17	1.07 (0.51-2.26)	0.852
Sleep disturbance	16	1.52	9,844	0.16	1.18 (0.67-2.09)	0.563

^a^ Abbreviation: IR, incident rate per 1,000 person-year; NMOSD, neuromyelitis optica spectrum disorder; CCI, Charlson comorbidity index.

**Table 4:** Subgroup analysis by the severity of neuromyelitis optica spectrum disorder.

Variables	Peripheral artery disease		Venous thromboembolism					
			Overall		Deep venous thrombosis		Pulmonary embolism	
	aHR (95% CI) a	p-value	aHR (95% CI) a	p-value	aHR (95% CI) a	p-value	aHR (95% CI) a	p-value
NMOSD cohort	1.40 (1.02-1.92)	0.039	4.81 (3.08-7.52)	<0.001	8.22 (4.55-14.88)	<0.001	2.29 (1.10-4.77)	0.027
By the total length of hospital stay								
Mild	1.50 (0.99-2.27)	0.058	1.31 (0.51-3.32)	0.574	1.76 (0.52-5.97)	0.367	0.90 (0.21-3.85)	0.882
Moderate	0.49 (0.20-1.19)	0.116	5.05 (2.58-9.87)	<0.001	7.64 (3.17-18.42)	<0.001	3.78 (1.42-10.06)	0.008
Severe	2.26 (1.41-3.61)	<0.001	11.69 (6.86-19.90)	<0.001	23.83 (12.20-46.51)	<0.001	3.13 (1.06-9.20)	0.038

^a^ aHR, adjusted hazard ratio. Adjustment for sex, age, insured salary, urbanization, CCI score, and all comorbidities.

**Table 5:** Stratified analysis of the risk of peripheral artery disease and venous thromboembolism.

Variables	NMOSD ^a^ vs Comparison (ref.)				
	Adjusted HR ^b^	95% CI			p-value
Peripheral artery disease					
Sex					
Female	1.25	0.86	-	1.81	0.244
Male	1.97	1.04	-	3.74	0.039
Age (year)					
<25	2.24	0.41	-	12.36	0.354
25-34	2.55	0.89	-	7.31	0.081
35-44	1.85	0.92	-	3.73	0.086
≥45	1.17	0.79	-	1.74	0.436
Venous thromboembolism					
Sex					
Female	5.21	3.21	-	8.46	<0.001
Male	2.80	0.84	-	9.33	0.093
Age (year)					
<25	-		-		-
25-34	7.98	1.52	-	41.85	0.014
35-44	9.01	3.01	-	26.97	<0.001
≥45	3.51	2.01	-	6.12	<0.001

^a^ Abbreviation: NMOSD, neuromyelitis optica spectrum disorder. ^b^ The adjustment variables contained sex, age, insured salary, urbanization, CCI score, and related comorbidities.

## Data Availability

Datasets from the National Health Insurance Research Database (NHIRD) were retrieved in this retrospective cohort study, and the data are available from the Taiwan National Health Insurance (NHI) Bureau. The data is not publicly available because of legal restrictions regarding the “Personal Information Protection Act” in Taiwan. However, requests for data can be formally sent to the NHI bureau. (https://dep.mohw.gov.tw/DOS/cp-2516-3591-113.html).
